# *Candidatus* Liberibacter asiaticus accumulation in the phloem inhibits callose and reactive oxygen species

**DOI:** 10.1093/plphys/kiac346

**Published:** 2022-07-26

**Authors:** Chiara Bernardini, Donielle Turner, Chunxia Wang, Stacy Welker, Diann Achor, Yosvanis Acanda Artiga, Robert Turgeon, Amit Levy

**Affiliations:** Citrus Research and Education Center, University of Florida, Lake Alfred, Florida 33850, USA; Citrus Research and Education Center, University of Florida, Lake Alfred, Florida 33850, USA; Citrus Research and Education Center, University of Florida, Lake Alfred, Florida 33850, USA; Citrus Research and Education Center, University of Florida, Lake Alfred, Florida 33850, USA; Citrus Research and Education Center, University of Florida, Lake Alfred, Florida 33850, USA; Citrus Research and Education Center, University of Florida, Lake Alfred, Florida 33850, USA; School of Integrative Plant Science, Plant Biology Section, Cornell University, Ithaca, New York 14853, USA; Citrus Research and Education Center, University of Florida, Lake Alfred, Florida 33850, USA; Department of Plant Pathology, University of Florida, Gainesville, Florida 32611, USA

## Abstract

CLas inhibits callose deposition in the sieve pores and the accumulation of reactive oxygen species to favor its cell-to-cell movement.

Dear Editor,

Huanglongbing (HLB) is a severe disease in citrus that is associated with *Candidatus* Liberibacter asiaticus (*C*Las). The study of *C*Las–phloem interaction has been hampered because of the low and unequal distribution of *C*Las inside trees, and the difficulty in isolating vasculature from trees. Previous studies reached the conclusion that infection increases sieve pores callose levels and the production of H_2_O_2_, blocking the phloem and causing a toxic build-up leading to programmed death (Achor et al., 2010; Pitino et al., 2017; Deng et al., 2019; Welker et al., 2021; Ma et al., 2022), but even so the bacteria can still move in the phloem. To better understand *C*Las–phloem interactions, we used the isolated vasculatures of “Hamlin” sweet orange (*Citrus sinensis*) and “Duncan” grapefruit (*Citrus paradisi*) seeds, which highly accumulate *C*Las (Figure 1A). We show that *C*Las inhibits plant cellular defense responses to allow its movement through the sieve pores.

**Figure 1:**
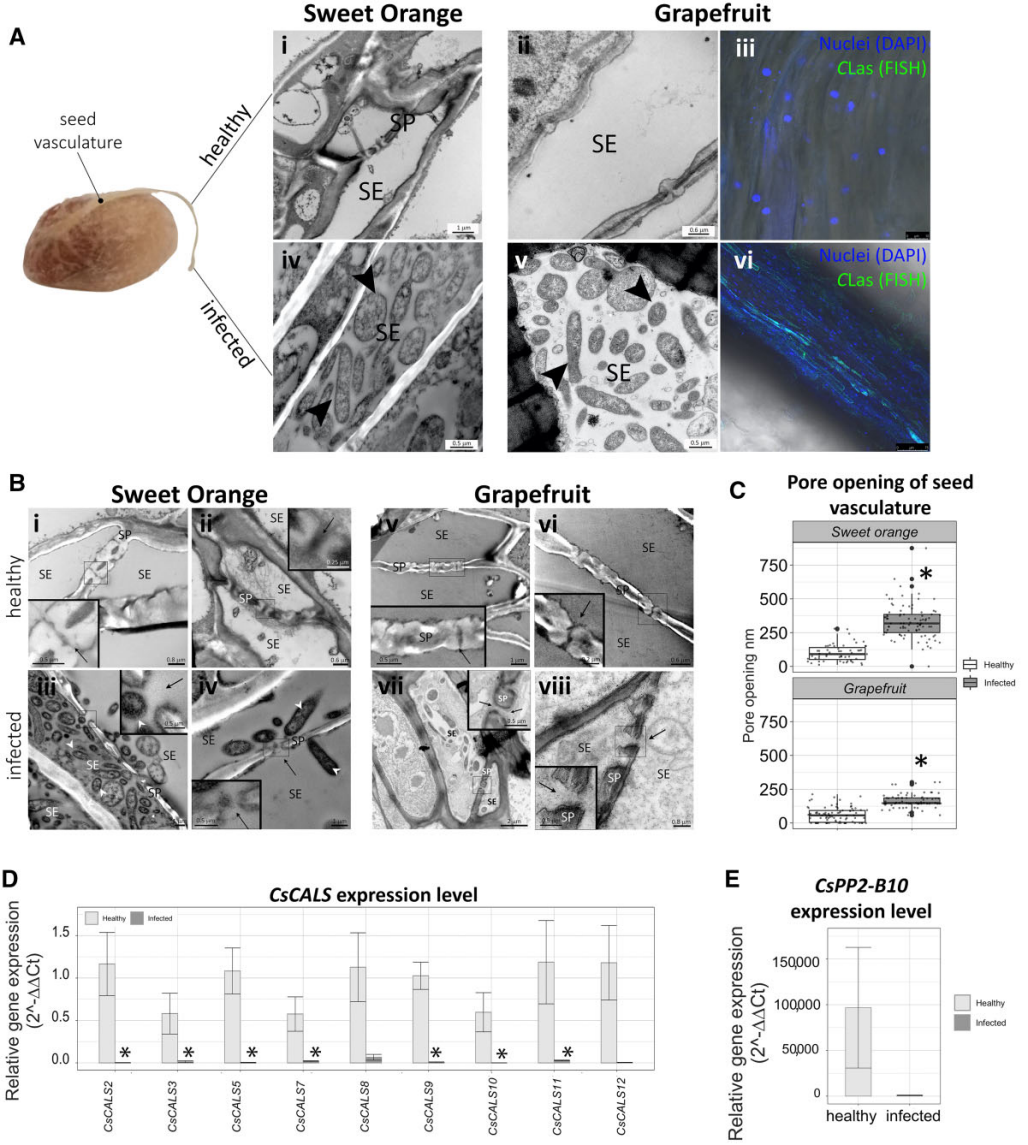
*C*Las accumulates in seed vasculature and reduces phloem plugging. A, *C*Las accumulates in the seed coat vasculature (i–ii; iv–v): Transmission electron microscope (TEM) micrographs of seed vasculature of “Hamlin” Sweet Orange (i; iv) and “Duncan” grapefruit (ii; vi) from HLB trees (infected) or trees grown under protective screen (healthy). triangles = *C*Las. (iii; vi): fluorescent in situ hybridization (FISH) micrographs of healthy and infected seed vasculatures of “Duncan” grapefruit (iii–vii). Nuclei are labeled with 4′,6-diamidine-2′-phenylindole dihydrochloride (DAPI) and *C*Las labeled with a FISH probe. Bars in i, ii =0.6 µm; iv, v = 0.5 µm; iii = 10 µm, and vi = 7.5 µm. B, Micrographs of healthy (i and ii) and infected (iii and iv) seed vasculatures of “Hamlin” sweet orange (bar = 1, 0.6, 1, and 1 µm, respectively) and micrographs of healthy (v and vi) and infected (vii and viii) seed vasculatures of “Duncan” grapefruit (bar = 1, 0.6, 2, and 0.8 µm, respectively). Arrows = sieve pores, triangles = *C*Las. C, Sieve plate pore size in “Hamlin” sweet orange and “Duncan” grapefruit. The lower end of the whiskers shows the minimum value and the upper end of the whiskers shows the maximum value. The lower bottom of the box represents the 25 percentile of data from the smallest, the horizontal line in the box the median and the upper bottom the 75th percentile. Larger points are outliers. Asterisk expresses significant differences among the means (*n* = 85 and 83 sieve pores of healthy and infected “Duncan” Grapefruit, respectively, and 102 and 87 of healthy and infected “Hamlin” Sweet Orange, respectively), with *P* ≤ 0.05 (Student’s *t* test). D, Relative abundance of *CsCalS* gene transcripts in healthy and infected seed vasculature of “Duncan” grapefruit. E, Relative abundance of *CsPP2-B10* gene transcripts in healthy and infected seed vasculature of “Duncan” grapefruit. Data are expressed as mean ± SE of four independent biological replicates (total four trees, each replicate is a pool of seed vasculatures from one tree). Differences among healthy and infected means were evaluated with Student’s *t* test. Asterisks represent significant differences at *P* ≤ 0.05.

Callose is a polymer of β-1,3 glucan, synthetized by callose synthases, that forms structural components of plant cells (Ellinger and Voigt, 2014). In infected leaves callose accumulated in the sieve pores, the expression of *C. sinensis* CalS and phloem protein 2 (*CsPP2*) genes increased, and the phloem pore opening decreased, but these sieve elements (SEs) had very low levels of *C*Las (Granato et al., 2019; Achor et al., 2020). We examined SEs in infected leaves, and observed *C*Las, on average, in only ∼7.76% of the SEs (Supplemental Table S1). While we cannot rule out the presence of *C*Las in more SEs because of the thinness of the sections, these results indicate that *C*Las levels in leaves are low and suggest that the extensive callose and H_2_O_2_ accumulation are mainly plant immune responses. In sink tissues, *C*Las was present in higher numbers (suggesting *C*Las predominantly traffics along with phloem translocation) and callose levels were lower (Achor et al., 2020). In seed vasculatures sieve plate (SP) pores were not occluded by callose (Achor et al., 2020; this study). To determine whether this resulted from a lack of callose deposition in the seeds, or from the presence of *C*Las, we compared infected and uninfected seed vasculature (Supplemental materials and methods). Thick deposits of callose were present at the SPs of healthy sweet orange and grapefruit seed vasculatures (Figure 1B). In infected seeds, SEs lumen was filled by bacteria and no callose layer was visible inside the pores. Pore opening increased significantly (Figure 1C) and the expression of *CsCalS* and *CsPP2* genes was downregulated (Figure 1, D and E).

To assess if the lack of callose is related with the seed vasculature or is a general activity of the pathogen, we examined the sieve pores in young leaf midribs from infected trees (Figure 2A). In the cells that contained *C*Las, the pore diameter increased compared to the cells without *C*Las (Figure 2, A and B). These results indicate that *C*Las either inhibits the deposition of callose or induces its removal in both leaves and seeds. The reduction of the callose layer increased the available cytoplasmic space in the sieve pores and consequently we could observe *C*Las crossing the SPs (Supplemental Figure S1). This activity resembles the movement protein of viruses that also inhibit the deposition of callose to facilitate movement through plasmodesmata (Rojas et al., 1997; Guenoune-Gelbart et al., 2008; Wang, 2021).

**Figure 2:**
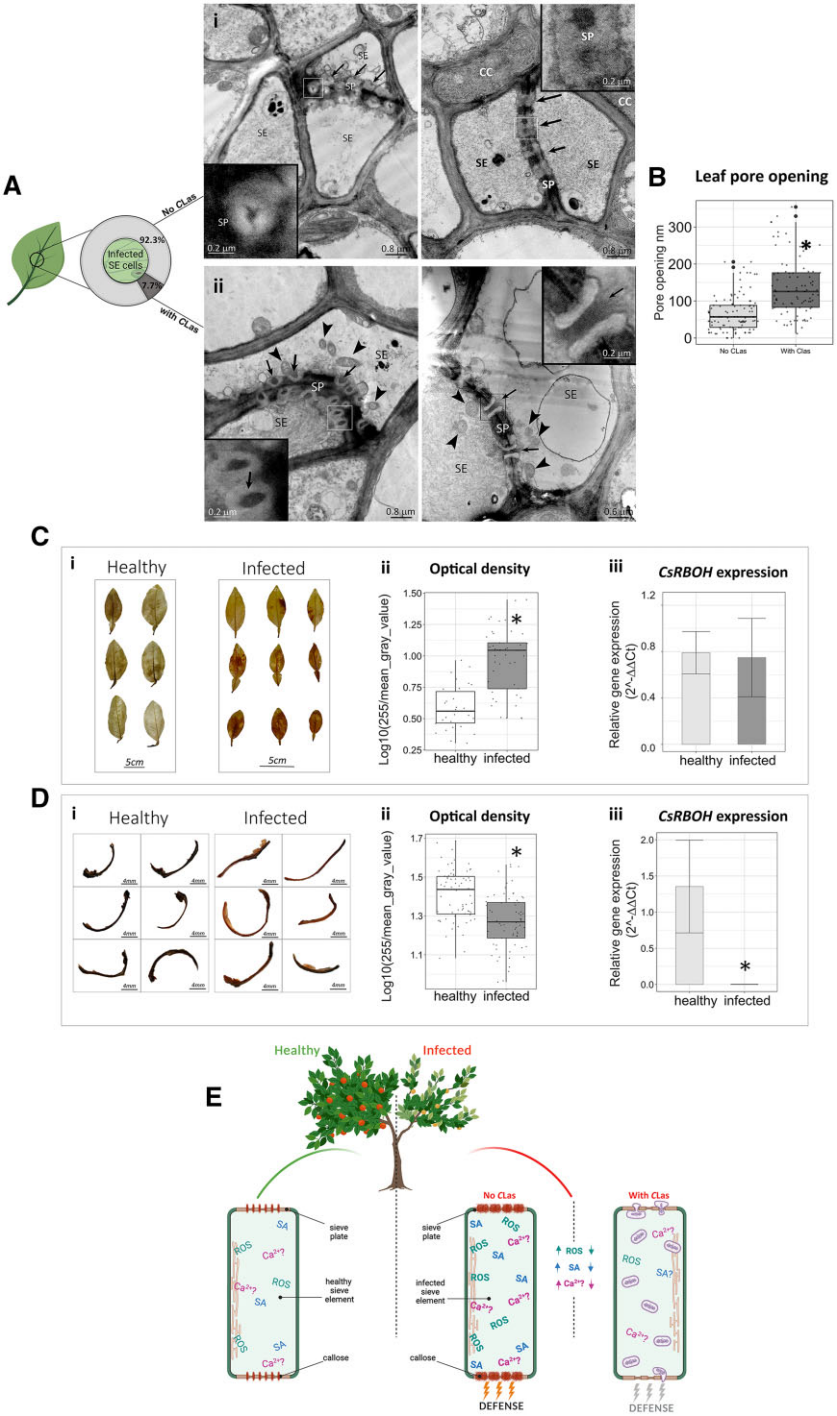
*C*Las Inhibits plant defense response. A, Percentage of cells without *C*Las- or with *C*Las in midrib of HLB-infected plant, and micrograph of sieve plates in *C*Las-free (i; bars = 0.8 µm) or *C*Las-containing (ii; bars = 0.8 and 0.6 µm) cells, all from infected trees. Arrows = sieve pores, triangles =*C*Las, CC = companion cells. B, Pore opening value in *C*Las-free and *C*Las-containing cells. The boxplot reports the pore opening in nm. Asterisk expresses significant differences among the means (*n* = 106 sieve pores of *C*Las-free SEs and 92 pores in SE with *C*Las), with *P* ≤ 0.05 (Student’s *t* test). The lower end of the whiskers shows the minimum value and the upper end of the whiskers shows the maximum value. The lower bottom of the box represents the 25th percentile of data from the smallest, the horizontal line in the box the median and the upper bottom the 75th percentile. Larger points are outliers. Picture created with BioRender software (BioRender.com, 2022). C, “Duncan” grapefruit leaves stained with DAB from healthy and infected plants (i). Bar = 5 cm. Optical density of healthy and infected leaves (five healthy and five infected trees; at least six leaves were randomly selected in each plant) (ii). Asterisk expresses significant differences among the means (Student’s t test, *P* ≤ 0.05). *CsRBOH* leaf expression level in “Duncan” grapefruit (iii), expressed as mean ± SE of four independent biological replicates (each replicate is a pool of three leaf midribs from one tree). Asterisk expresses significant differences among the means (Student’s t test, *P* ≤0.05). D, “Duncan” grapefruit vasculatures stained with DAB (i) from healthy and infected seeds. Bar = 4 mm. Optical density of healthy and infected seed vasculatures (ii). Asterisk expresses significant differences among the means (at least 10 seed vasculatures were extracted from 3 fruits chosen randomly from each of 5 healthy and 5 infected trees) (Student’s *t* test, *P* ≤ 0.05). *CsRBOH* seed vasculature expression level (iii) in “Duncan” grapefruit seed vasculatures expressed as mean ± se of four independent biological replicates (each replicate is a pool of seed vasculatures from one tree). Asterisk denotes significant differences among the means (Student’s *t* test, *P* ≤ 0.05). E, Model for *C*Las–phloem interaction in HLB-infected trees. In healthy SEs, a physiological level of ROS , salicylic acid (SA), and Ca^2+^ is present in the phloem sap. Around the sieve pores, a normal physiological layer of callose ensures proper transport of substances through the phloem. In infected SE cells without *C*Las, the ROS and SA contents increase. Ca^2+^ may increase as well. Callose completely occludes the sieve pores. In *C*Las-containing SE cells, ROS concentration decreases. The concentration of Ca^2+^ and SA probably decrease as well. Sieve pore callose is completely absent, allowing the movement of the bacteria. Ca^2+^ = calcium ions. Picture created with BioRender software (BioRender.com, 2022).

Previous research demonstrated that reactive oxygen species (ROS) were also elevated in *C*Las-infected leaves (Pitino et al., 2017; Ma et al., 2022). We measured the intensity of 3,3'-diaminobenzidine (DAB) staining (which turns red in the presence of H_2_O_2_) in leaves and seed vasculatures from healthy and infected samples (Supplemental materials and methods). Infected leaves had more stained areas, indicating the production of H_2_O_2_ (Figure 2C). In the seed vasculatures, H_2_O_2_ levels were lower in infected samples compared to the healthy ones (Figure 2D). The RBOH protein is the provider of $O_{2}^{-}$ ions, a ROS (Pitino et al., 2017). In the seed vasculature, the *CsRBOH* gene was downregulated in case of infection (Figure 2D (iii)), while no significant modulation of *CsRBOH* occurred in the leaves (Figure 2C (iii)). The contrasting results between leaves and seed vasculatures (in which *C*Las is present at much higher numbers) indicate that *C*Las inhibits H_2_O_2_ production inside cells.

Results from this work are summarized in Figure 2E. Healthy plant SEs have a normal layer of callose around the sieve pore and physiological level of ROS. In infected SEs that do not contain bacteria, there is an increase of callose and ROS. In cells where *C*Las accumulates, callose and ROS are reduced, allowing the bacteria to survive and move. We hypothesize that the callose and ROS accumulation may result from increased Ca^2+^ and SA (Nehela et al., 2018; Liu et al., 2019). The pathogen may locally subtract Ca^2+^ (Liu et al., 2019), reduce ROS toxicity (Jain et al., 2015), and hydrolyze SA (Li et al., 2017). *C*Las-secreted peroxiredoxin was shown to suppress the plant immune response, including callose and ROS (Jain et al., 2018). HLB disease symptoms appear to result from a failed strategy on the part of the citrus host, where the cost of defense outweighs the damage done by the pathogen itself. Analysis of the *C*Las genome reveals an organism with largely defensive capabilities, making sense for an obligate parasite (Duan et al., 2009). Blocking the attempts of *C*Las to bypass the plant defenses can provide a strategy for eliminating the bacteria and establish resistant varieties. Paradoxically, the bacterial activities that counteract the host defenses may also alleviate some disease symptoms. Plants which have less callose expression have shown better resistance to powdery mildew (Jacobs et al., 2003; Nishimura et al., 2003). This phenomenon has not been studied in the context of phloem bacterial pathogens. Considering this information, a “non-confrontational” strategy for the development of HLB-tolerant citrus might also be considered. Citrus stocks which naturally exhibit low callose and ROS responses to *C*Las seem to remain free from the symptoms (Deng et al., 2019; Curtolo et al., 2020). This strategy might also be examined in other plant–pathogen interactions which involve bacteria inhabiting the vascular system. Future work is required to explore this possibility.

## Supplemental data

The following materials are available in the online version of this article.

**Supplemental Figure S1.** *C*Las passage through open sieve pores in seed vasculatures.

**Supplemental Table S1.** Percentage of SE containing *C*Las in young leaves (2-year observation).

**Supplemental materials and methods.**

## Supplementary Material

kiac346_Supplementary_Data

## Notes

A.L. conceived the project. C.B., C.W., and D.T. collected the samples. C.B. performed the ROS analyses and the statistical analysis. C.B. and D.T. carried out the gene expression analysis. D.A. and C.W. embedded the samples and visualized by TEM. Y.A.A. performed the fluorescent in situ hybridization analysis. A.L. and C.B. performed the imaging analyses. C.B. and A.L. wrote the manuscript. R.T. revised the article. S.W. edited the article. All the coauthors provided critical comments for the text of the manuscript.

The author responsible for distribution of materials integral to the findings presented in this article in accordance with the policy described in the Instructions for Authors (https://academic.oup.com/plphys/pages/general-instructions) is Amit Levy (amitlevy@ufl.edu).
