# Supplementary material for: Candidatus Liberibacter asiaticus accumulation in the phloem inhibits callose and reactive oxygen species
Source: Plant Physiol. 2022 Jul 26;190(2):1090–4. doi: 10.1093/plphys/kiac346 (PMC9516723; doi:10.1093/plphys/kiac346)
Supplement: kiac346_Supplementary_Data [file kiac346_supplementary_data.pdf]

Supplemental Figure S1:

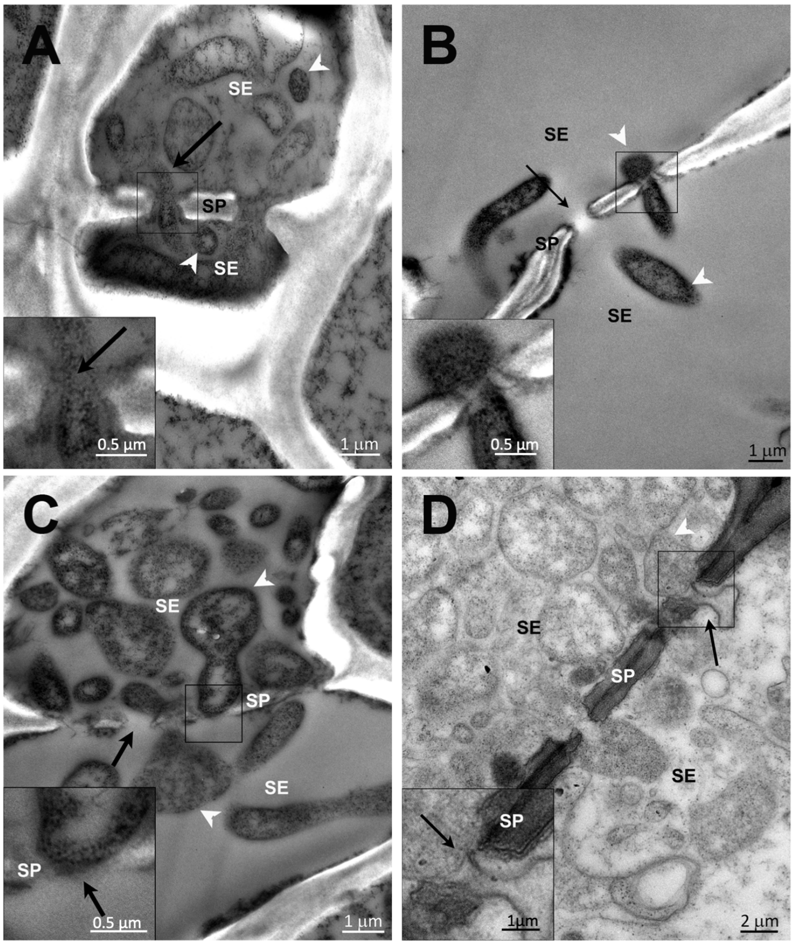

***C*Las passage through open sieve pores in seed vasculatures.** Sieve pores of sweet orange (**A-C**) and grapefruit (**D**) with *C*Las. Label: SE= sieve element, SP=sieve plates, triangles= *C*Las. Arrows indicate the passage of *C*Las through the sieve pore without the callose layer. Bar= 1, 1, 1, and 2 µm respectively.

Supplemental Table S1: Percentage of SE containing *C*Las in young leaves (two-year observation) in both Sweet Orange and Grapefruit. The number of SE examined express the total amount of sieve elements observed with a TEM (Morgagni 268 transmission electron microscope) belonging to plants tested positive. Number of SE containing CLas expresses only the sieve element cells with at least one living bacteria.

Citrus variety	SE examined	SE containing CLas	SE containing CLas (%)
Sweet Orange -I	36	5	13.89
Sweet Orange-II	65	5	7.69
Grapefruit-I	107	2	1.87
Grapefruit-II	6	0	0

Supplemental Materials and Methods

Sample collection

Fruits and flush leaves of *Candidatus* Liberibacter asiaticus (*C*Las)-infected ‘‘Hamlin’ sweet orange (*Citrus sinensis* L.) and ‘Duncan’ grapefruit (*Citrus* x *paradisi*) (DG) were collected simultaneously in experimental fields in Polk County (July 2020) and Collier County (July 2021), Florida, USA at the fully symptomatic stage. Healthy fruits and leaves belonging to the same varieties were collected at the same time from plants grown under protective screens. Healthy samples were used as a control group.

Seeds were collected from fruit stored at 4°C until seed vasculature extraction. The testa and the tegmen of the seed were removed with forceps to expose the vascular tissue of the embryo. The vasculatures were carefully removed from the apex at the point of junction with the embryo. The extracted vasculatures were used either immediately (microscopy analysis), stored at 4°C for staining, or stored at -20°C for molecular analysis.

Electron microscopy and imaging analysis

Electron microscopy analysis was performed as previously described (Achor et al., 2020; Folimonova and Achor, 2010), using a standard fixation procedure as follows. Midrib samples (0.5mm of length) and seed vasculature samples (whole vasculature), were collected from 3 different plants. Samples were fixed with 3% (v/v) glutaraldehyde in 0.1 M of potassium phosphate buffer at pH 7.2 for 4 h at room temperature, washed in phosphate buffer, then postfixed in 2% osmium tetroxide (w/v) in the same buffer for 4 h at room temperature. The samples were further washed in the phosphate buffer, dehydrated in a 10% acetone (v/v) series (10 min per step), and infiltrated and embedded in Spurr’s resin over 3 d. Sections (100-nm) were mounted on 200-mesh formvar-coated copper grids, stained

with 2% aq uranyl acetate (w/v) and Reynold's lead citrate. Ultrathin sections of the samples were observed through a Morgagni 268 transmission electron microscope (TEM). Pictures obtained from the TEM observations were analyzed with FIJI. For each sieve plate, the opening of the pores was measured, and analyzed as reported below.

Confirming the accumulation of CLas in the seed vasculature

The accumulation of the bacteria in the seed vasculature was assessed with fluorescent in situ hybridization following a previously reported protocol (Ghanim et al., 2009) and adapted for use with plant tissue (Hilf et al., 2013). Samples were visualized with a Leica SP8 laser-scanning confocal microscope (Leica Microsystems Inc., Buffalo Grove, IL) with a 488nm argon excitation laser for Alexa Fluor 488 and a near UV diode 405nm for DAPI (4′,6-Diamidine-2′-phenylindole dihydrochloride). Emission signals were respectively detected at 520nm and 480nm with a gain of 600.

H_2O_2 concentration assay

Samples of leaves were collected from the field and immediately placed in a solution of 1mg/ml 3,3'-diaminobenzidine (Sigma-Aldrich) (DAB) in water, pH 3.8, prepared as previously reported (Daudi and O'Brien, 2012; Kumar et al., 2014). Seed vasculatures collected from the fruits, stored at 4°C, were placed in DAB solution. The staining protocols were performed as follows: overnight staining in DAB on a shaker at 50 rpm. The following day destain for 20 minutes (for leaf samples) or 10 minutes (for seed vasculature samples) in a bleaching solution with the composition of ethanol:glycerol:acetic acid in a 3:1:1 ratio at 92°C (Daudi and O'Brien, 2012; Kumar et al., 2014). After the initial bleaching, the solution was replaced with fresh bleaching solution and the samples were left at room temperature for 30 minutes before the observation. Leaf samples were collected from 10 plants (5 healthy and 5 infected) and at least 6 leaves selected randomly in each plant were

observed. For seed vasculature analysis, seed vasculatures were extracted from 3 fruits chosen randomly from each of 10 plants (5 healthy and 5 infected). For each fruit at least 10 seed vasculatures were observed, and pictures were acquired with a stereo microscope Leica KL300 on a white background (Leica Microsystem 2021) and using the LASX software (Leica Application Suite, Leica microsystem 2021). For each picture the same exposure time, light and white balance conditions were applied.

Leaf pictures were analyzed with FIJI software (Schindelin et al., 2012). On each picture, color deconvolution was performed with the H DAB algorithm (Crowe and Yue, 2019). The channel corresponding to the DAB color was separately saved and used for the analysis. For each leaf the mean gray value (intensity of the light) of the whole lamina was analyzed, extracting the lamina from the background.

Vasculature tissue was analyzed with the workflow as described above. For each vasculature, 5 regions of interest (ROIs), each 576 pixels squared in size, were chosen randomly to avoid readings affected by the size of the vasculature. For each ROI, the mean gray value was recorded.

The mean gray value was transformed to optical density value with the following formula: log(max intensity / mean intensity) in which the maximum intensity for 8-bit pictures has a value of 255 (Nguyen and Nguyen, 2013).

Gene expression analysis

Total RNA extraction on Duncan seed vasculatures and leaf midribs were performed using Trizol reagent (Invitrogen). RT-qPCR was performed as previously described (Achor et al., 2010), starting from roughly 100 mg of fresh tissue. RNA reverse transcription was carried out using a High-Capacity cDNA Reverse Transcription kit (Applied Biosystems), following the manufacturer instructions starting from 500 ng of RNA. To determine difference in modulation of the genes, RT-

PCR was performed using SYBR Green FastMix (Quantabio, Beverly, MA, USA) starting from 300 ng of cDNA in a total reaction volume of 15 µL with a concentration of each primer of 400 nM. All gene primers used for RT-qPCR are listed in Table 1. Gene expression was compared to the citrus GAPDH reference gene, and analysis was performed using the $2^{-\Delta\Delta Ct}$ method (Livak and Schmittgen, 2001).

Table 1: List of the genes analyzed in this study and relative primers used to amplify them.

Gene	Primer sequence 5'-3'	Source
CsRBOH	Sense CCCTCGGCTTATAAATGCAA<br>Antisense CAAAAGGCATTGAACCCAGT	Pitino et al., 2017
CsCALS2	Sense ATCTCTGCCGGTTCTATGCG<br>Antisense CGGGCATCACTCTTTGACCT	Granato et al., 2019
CsCALS3	Sense GGCCTCCGTTCTTACTTGCT<br>Antisense ACACTCCTTGACAGCACAGG	Granato et al., 2019
CsCALS5	Sense GTGTGATTGAAACG GAAGCCA<br>Antisense CCATCATCACGCATAGGCCA	Granato et al., 2019
CsCAL7	Sense GACGCCTAACCGAGTACCTGC<br>Antisense GTGCAGCTGGTGATCCATCA	Granato et al., 2019
CsCALS8	Sense AGGATGTTTTCGCCGGTACA<br>Antisense ATCACGACCTTTGCCCACTT	Granato et al., 2019
CsCALS9	Sense TCCTTTCTCGAATTGGCCGT<br>Antisense TGTCTGTGCGCGATATGAGG	Granato et al., 2019
CsCAL10	Sense GGCTCGACTTGGCATACCTG<br>Antisense AACTGTTCCAAGCAAGGCGT	Granato et al., 2019
CsCALS11	Sense GATGTGTACCGCTT GGGTCA<br>Antisense AGCAAGATAAAGACGCCCCC	Granato et al., 2019
CaCALS12	Sense CTTGGGTCAGCGTGTTTTGG<br>Antisense CTCCTCGCAGTGTGCAGTTA	Granato et al., 2019
GAPDH	Sense TGGCGACCAAAGGCTACTC<br>Antisense TTGCCGCACCAGTTGATG	Mafra et al., 2012

Data analysis

Statistical analyses were performed using R with Rstudio software Version 1.1.456 (RStudio Team (2020). RStudio: Integrated Development for R. RStudio, PBC, Boston, MA). Conformity to the normal distribution and homogeneity of variances were checked with Shapiro-Wilk's test and Bartlett's test respectively. Where necessary, data were normalized with a Box-Cox transformation. For each analysis, Student's t-test was used to determine significant differences among the treatment group means (healthy or infected) with $p < 0.05$.

Achor, D., Etxeberria, E., Wang, N., Folimonova, S., Chung, K., Albrigo, L., 2010. Sequence of anatomical symptom observations in citrus affected with huanglongbing disease. Plant Pathol J 9, 56–64.

Achor, D., Welker, S., Ben-Mahmoud, S., Wang, C., Folimonova, S.Y., Dutt, M., Gowda, S., Levy, A., 2020. Dynamics of *Candidatus* Liberibacter asiaticus Movement and Sieve-Pore Plugging in Citrus Sink Cells. Plant Physiol. 182, 882–891. https://doi.org/10.1104/pp.19.01391

Crowe, A., Yue, W., 2019. Semi-quantitative Determination of Protein Expression Using Immunohistochemistry Staining and Analysis: An Integrated Protocol. BIO-PROTOCOL 9. https://doi.org/10.21769/BioProtoc.3465

Daudi, A., O'Brien, J., 2012. Detection of Hydrogen Peroxide by DAB Staining in Arabidopsis Leaves. BIO-PROTOCOL 2. https://doi.org/10.21769/BioProtoc.263

Folimonova, S.Y., Achor, D.S., 2010. Early Events of Citrus Greening (Huanglongbing) Disease Development at the Ultrastructural Level. Phytopathology® 100, 949–958. https://doi.org/10.1094/PHYTO-100-9-0949

Ghanim, M., Brumin, M., Popovski, S., 2009. A simple, rapid and inexpensive method for localization of Tomato yellow leaf curl virus and Potato leafroll virus in plant and insect vectors. Journal of virological methods 159, 311–314.

Granato, L.M., Galdeano, D.M., D'Alessandre, N.D.R., Breton, M.C., Machado, M.A., 2019. Callose synthase family genes plays an important role in the Citrus defense response to Candidatus Liberibacter asiaticus. Eur J Plant Pathol 155, 25–38. https://doi.org/10.1007/s10658-019-01747-6

Hilf, M.E., Sims, K.R., Folimonova, S.Y., Achor, D.S., 2013. Visualization of ' *Candidatus* Liberibacter asiaticus' Cells in the Vascular Bundle of Citrus Seed Coats with Fluorescence In Situ Hybridization and Transmission Electron Microscopy. Phytopathology® 103, 545–554. https://doi.org/10.1094/PHYTO-09-12-0226-R

Kumar, D., Yusuf, M., Singh, P., Sardar, M., Sarin, N., 2014. Histochemical Detection of Superoxide and H2O2 Accumulation in Brassica juncea Seedlings. BIO-PROTOCOL 4. https://doi.org/10.21769/BioProtoc.1108

Livak, K.J., Schmittgen, T.D., 2001. Analysis of relative gene expression data using real-time quantitative PCR and the $2^{-\Delta\Delta CT}$ method. methods 25, 402–408.

Mafra, V, Kubo, K. S, Alves-Ferreira, M, Ribeiro-Alves, M, Stuart, R. M, Boava, L. P, Rodrigues, C.M, Machado, M. A (2012). Reference genes for accurate transcript normalization in citrus genotypes under different experimental conditions. PloS one, 7(2), e31263.

Nguyen, D., Nguyen, D., 2013. Quantifying chromogen intensity in immunohistochemistry via reciprocal intensity. Protocol Exchange. https://doi.org/10.1038/protex.2013.097

Pitino, M, Armstrong, C.M, Duan, Y, (2017) Molecular mechanisms behind the accumulation of ATP and H2O2 in citrus plants in response to 'Candidatus Liberibacter asiaticus' infection. Hortic Res 4, 17040. https://doi.org/10.1038/hortres.2017.40

Schindelin, J., Arganda-Carreras, I., Frise, E., Kaynig, V., Longair, M., Pietzsch, T., Preibisch, S., Rueden, C., Saalfeld, S., Schmid, B., Tinevez, J.-Y., White, D.J., Hartenstein, V., Eliceiri, K.,

Tomancak, P., Cardona, A., 2012. Fiji: an open-source platform for biological-image analysis. Nature Methods 9, 676–682. https://doi.org/10.1038/nmeth.2019
